# Integrating Mobile health and Physical Activity to reduce the burden of Chronic low back pain Trial (IMPACT): a pilot trial protocol

**DOI:** 10.1186/s12891-015-0852-3

**Published:** 2016-01-19

**Authors:** Anita B. Amorim, Evangelos Pappas, Milena Simic, Manuela L. Ferreira, Anne Tiedemann, Matthew Jennings, Paulo H. Ferreira

**Affiliations:** Discipline of Physiotherapy, Faculty of Health Sciences, The University of Sydney, 75 East Street, Lidcombe, Sydney, NSW 1825 Australia; The George Institute for Global Health, Sydney Medical School, The University of Sydney, Sydney, NSW Australia; Institute of Bone and Joint Research, The Kolling Institute, Sydney Medical School, The University of Sydney, Sydney, NSW Australia; Physiotherapy Department, Liverpool Hospital, South Western Sydney Local Health District, Sydney, NSW Australia

**Keywords:** Physical activity, Low back pain, Mobile health, Health coaching

## Abstract

**Background:**

It is well recognised that low back pain is a significant public health problem and engagement in moderate levels of physical activity is associated with positive outcomes.

Conservative active care, such as exercise, is effective in reducing pain and disability associated with chronic low back pain. However, a rapid decline in clinical outcomes is commonly seen after discharge from treatment.

**Methods/Design:**

We will conduct a randomised controlled trial to investigate the effectiveness of a mobile health supported physical activity intervention (compared to standard care) in care-seeking, pain and disability in people with chronic low back pain after discharge from treatment. We will recruit 68 patients with chronic low back pain following discharge from an outpatient hospital program, who will be randomly allocated to the physical activity intervention (*n* = 34) or the standard care group (*n* = 34) and monitored for 6 months. The physical activity intervention will involve a physical activity advice booklet, a face-to-face health coaching session and 12 fortnightly follow-up telephone-based health coaching sessions. This intervention will be supported by provision of a specifically designed web app and a physical activity monitoring device (*FitBit*). The standard care group will receive the physical activity advice booklet only.

**Discussion:**

This pilot trial will investigate a new model to prevent clinical decline in people following conservative treatment for chronic low back pain. If proven to be effective, this approach will constitute a major advance in the management of low back pain. Chronic patients who experience recurrent pain and disability after treatment are prone to seek additional care in the form of physiotherapy, medication, emergency department attendance, specialist consultation or spinal surgery. This model aims to maintain functional levels and reduce care-seeking empowering patients to self-manage their low back pain by offering them a contemporary patient-centred physical activity program with the support of mobile health technology. The outcomes of this trial will have immediate implications for clinical practice.

**Trial registration:**

ACTRN12615000189527 (26-02-2015).

## Background

It is well recognised that low back pain (LBP) is a significant public health problem [1]. Relapses in pain (60 %) and work absences (33 %) are common, making LBP one of the most costly conditions in industrialised societies [2]. The total cost associated with the management of chronic LBP in Australia is estimated at AU$9.17 billion, with the cost of indirect care and productivity losses contributing Ai1U$8.15 billion of this total figure [2]. Cost is expected to grow with the increasing obesity crisis and ageing population worldwide [3].

The clinical course of LBP is intricate. Over a 1-year after discharge from treatment, most patients will still have pain for a sustained period, and a small proportion will still have persistent severe pain [4, 5]. Although randomised controlled trials (RCT) investigating the efficacy of conservative interventions for chronic LBP have found improvements in pain and disability, patients usually exhibit a rapid decline in clinical outcomes, 3 months after treatment discharge [6, 7]. These patients are likely to have a new episode of LBP or continual pain and therefore seek additional health care [8].

Previous research has shown that patients with chronic LBP who engage in moderate to high levels of physical activity have better prognosis in terms of pain, disability, and quality of life than those who fail to maintain adequate levels of physical activity [9, 10]. However, ongoing adherence to such lifestyle behavior is difficult to achieve. Use of communication technologies to increase physical activity has become increasingly popular in recent years, likely because they facilitate access and adherence to health interventions [11]. The use of physical activity monitors is particularly effective in increasing engagement in physical activity [12]. New generations of activity monitors are affordable and provide feedback on daily steps taken and distance travelled using internet, tablet or Smartphone interfaces and social media. A recent systematic review provided strong evidence for the effectiveness of activity monitors such as pedometers to increase physical activity levels for patients with musculoskeletal disorders [13]. Additionally, physical activity monitors have been shown to not only improve physical activity levels, but also to increase function and reduce pain in patients with chronic LBP [14].

Another intervention that may be effective in improving adherence to exercise is health coaching. There is evidence that telephone-based health coaching interventions lead to positive changes in health behaviours. These include increased physical activity participation [14], improved nutrition [15], smoking cessation [16], and better management of chronic musculoskeletal conditions such as osteoarthritis [17]. Coaching interventions have a strong, evidence-based foundation in behaviour-change theories such as Social Influence Theory, Social Cognitive Theory, and the Transtheoretical Model [18] and can be effectively delivered by telephone [19]. There is evidence that health coaching via telephone has the potential to increase activity levels in patients with LBP when compared to usual physiotherapy care alone [20].

However, to the best of our knowledge, there are no studies of a post-treatment physical activity intervention consisting of mobile health and health coaching on long-term outcomes in patients with chronic LBP. Therefore, the primary aim of this RCT is to investigate the effec of a patient-centred physical activity intervention supported by health coaching and mHealth technology, including a mobile web app, tailored physical activity plan, goal setting, and feedback from affordable physical activity monitoring device (*FitBit*) in care-seeking, pain and disability in people with chronic LBP. Secondary outcomes will be physical activity participation and goal attainment. We hypothesise that the use of a patient-centred physical activity intervention will prevent clinical decline in patients who have received the benefits of conservative treatment for chronic LBP, empowering them to self-manage their LBP as well as to prevent worsening of LBP and reduce care seeking.

## Methods

### Study design

**Fig. 1:**
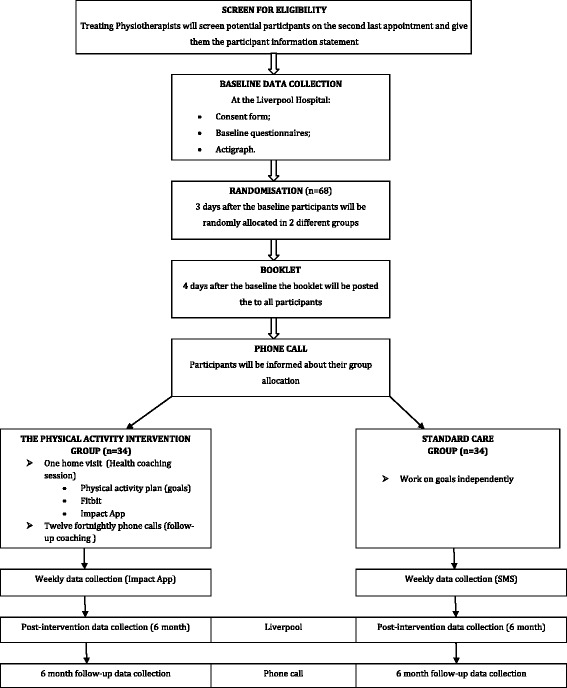
Flow diagram of the study

We will conduct a single-blinded pilot RCT to evaluate a patient-centered physical activity intervention involving health coaching, compared to standard care (Fig. 1). This trial has been designed according to the CONsolidated Standards Of Reporting Trials (CONSORT) statement [21] and is reported according to the Standard Protocol Items: Recommendations for Interventional Trials (SPIRIT) statement [22].

### Participants

We will recruit 68 patients with chronic LBP from an outpatient physiotherapy department in a public hospital in Sydney, Australia. Consenting participants will be randomly allocated to either the physical activity intervention group (*n* = 34) and receive a patient-centred physical activity promotion program involving health coaching, mHealth tools and advice booklet, or to a standard care group (*n* = 34) who will receive an advice booklet only.

#### Inclusion criteria

Adults over 18 years of age with chronic LBP persisting for over 12 weeks but without radicular symptoms; who have been discharged from a hospital-based, LBP physiotherapy program but still have consistent pain (at least 3 in the Numerical Rating Scale); with regular (weekly) use of a computer or internet-connected mobile/tablet device; and fluency in English (verbal and written).

#### Exclusion criteria

Any spinal surgery in the past 12 months; evidence of nerve root, spinal cord or caudal equine compression; severe spinal stenosis indicated by signs of neurogenic claudication (grade 3 to 4); fibromyalgia, or systemic/inflammatory disorder; comorbid health conditions that would prevent active participation in increasing physical activity levels: cardio-respiratory illnesses; LBP caused by involvement in a road traffic accident in the last 12 months or ongoing litigation; current or planned pregnancy.

### Recruitment method

Treating physiotherapists will screen (all) potential participants from the outpatient Physiotherapy Department of the Liverpool Hospital, South Western Sydney Local Health District, Australia, and inform them about the study. Potential participants interested in participating in the study will receive the Participant Information Statement and be referred to the research team. Patients with chronic LBP who have received any conservative physiotherapy treatment (e.g. exercises, spinal manipulative therapy) and meet the inclusion criteria will be invited to participate in the trial after treatment discharge. At treatment discharge, a research assistant will discuss the study and offer participation to those who meet the inclusion criteria. If they agree to participate a signed consent form will be recorded and baseline data will be collected.

### Group allocation

Random allocation to physical activity or standard care groups will occur after confirmation of eligibility and baseline assessment. Allocation will be blinded and performed using a computer-generated random allocation schedule operated by a remote researcher. The allocation of participants will be concealed by using sequentially numbered, sealed and opaque envelopes.

### Procedures

Patients attending outpatient physiotherapy will be screened by their treating physiotherapists, who will determine eligibility, inform about the trial objectives and invite participation. The ones that manifest interest will be given the Participant Information Statement by their treating physiotherapist and decide if they want to participate in the study. A senior physiotherapist from Liverpool Hospital will be responsible for booking potential participants on their second to last appointment to meet the investigator after discharge. At discharge, patients that agree to participate will be asked to sign the consent form. The assessor will collect demographic and anthropometric data (age, height, weight, waist circumference) as well as baseline data related to the study outcomes. A device able to accurately estimate how physically active a person is throughout the day (*Actigraph*) by measuring 3-dimensional body accelerations will be given to all participants at baseline with clear instructions for use and telephone support available. The *Actigraph* will collect accelerometer-based data over a 1-week period to account for day-to-day variation in physical activity levels [23]. Participants will be provided with pre-paid envelopes to return the devices to the research centres. The *Actigraph* has been successfully used to measure physical activity in large-scale population-based studies internationally [23, 24]. The analyses will be adjusted for accelerometer wear time defined by ‘off time’. Any period of greater than 60 min with no activity at all will be considered to be ‘off time’ and excluded from the analyses. Data will be extracted by a research assistant who will remain blinded to group assignment throughout the trial.

### Interventions

*The physical activity intervention group* will receive a booklet developed by Australia’s Department of Health called ‘*Make your move – Sit less, be active for life!’* The booklet includes information about physical activity and sedentary behaviour. Participants will also be advised to continue their usual activities. The physical activity intervention group will also receive an individualised patient-centred physical activity plan developed with the advice of a health coach. The focus of the patient-centred physical activity will be on a gradual increase in physical activity where participants will be encouraged to devise fortnightly goals to suit and advance their physical activity levels. This intervention will be supported by the use of mHealth, which will include a specifically designed mobile web application (web app) and a physical activity monitoring device (FitBit).

The intervention will address the following factors:

*Health coaching*: This will involve an initial individual face-to-face coaching session. The session can take up to 2 hours and it will be held at the participants’ home. The health coach will be an experienced physiotherapist with a health coaching certification. Their aim will be to motivate and support the participants to increase their physical activity levels. The health coaching session will encompass:

1. Increasing physical activity: Participants will be assisted to develop a physical activity plan that suits their lifestyle preferences. The World Health Organisation (WHO) guidelines addressing healthy adults recommend at least 150 min of moderate-intensity or 75 min of vigorous-intensity aerobic physical activity throughout the week or an equivalent combination of moderate- and vigorous-intensity activity. These recommendations relate not only to sports, but also to other leisure activities, work and transport activity [25]. Suitable local exercise opportunities will be identified using the NSW Ministry of Health’s *Active and Healthy* [26] online database.
2. Decreasing sedentary behaviour: Participants will be encouraged to increase incidental physical activity throughout the day. Options may include travelling by public transportation to work, walking to shops, carrying out a home exercise program, standing at work and spending less time sitting while at home.
3. Goal-Setting: Goals are more effective when they are important to the individual (e.g., self-set rather than assigned), realistic, can be monitored, and when the participant receives positive encouragement [27]. The health coach will jointly work with each participant to set short-term physical activity goals to be achieved fortnightly. The participants’ individual goals will be set taking into consideration the nature of LBP and its normal clinical course. Although the focus of the interaction between the coach and participants is not on symptom monitoring, if significant clinical decline (e.g. participants’ reports of nerve root compromise) is observed, coaches will advise participants to seek appropriate treatment. The health coach will aim to monitor their goals, giving them support and motivation.

This health coaching service will be modelled after the successful [19] NSW Ministry of Health initiative *Get Healthy* service. After the first individual face-to-face coaching session, the health coach will contact each participant fortnightly (12 phone calls over a 6 month period each participant) to assess progress, update the participants’ short-term goals and assist in overcoming barriers – e.g. how to get back to exercise after illness, and provide problem-solving strategies for maintaining physical activity.

*The Fitbit activity monitor and feedback device:* The activity monitoring device will be provided to the participant by the health coach during the initial visit who will also demonstrate its use. Each participant will receive a brief orientation covering this instrument’s proper functions. Telephone support will be offered to participants who have difficulty with the device setup. The *Fitbit* is a personal accelerometer device designed to track physical activity and sleep. This device logs individual data on physical activity and provides feedback on the number of steps taken in each 24-hour period; the distance walked daily; calories burned; and sleep duration and quality. The number of steps taken per day, week, and month are also summarised graphically on the device’s website (http://www.fitbit.com/au) or a Smartphone application, which will also be demonstrated to the participant.

*The IMPACT web app:* The IMPACT web app: A mobile web app will be built and hosted by the University of Sydney. The app will be customised specifically for the purposes of this project to allow participants to monitor their goals and their physical activities. Participants will be able to access the app at any time and write reports about their physical activity related goals to their health coach as well as receive coach-tailored feedback. In addition, each participant will receive a quick questionnaire every week to monitor their pain levels, based on the Numerical Rating Scale; disability, based on the Rolland-Morris disability questionnaire and care-seeking associated with LBP. This questionnaire was created specifically to this project. The health coach will have the ability to view the participant’s report and communicate with them by phone fortnightly to discuss their goals and update them according to their reports and feedback. Personalised messages constructed by the health coach will be sent every week to encourage participants to achieve their goals. In summary, the IMPACT web app will be designed to facilitate monitoring achievement and providing updates on participants’ goals, encouraging them to engage in physical activity and track potential adverse events.

*The standard care control group* will receive the ‘*Make your move – Sit less, be active for life!’* booklet and will be advised to work towards increasing their physical activity levels and achieving their two long-term goals as defined at baseline.

### Outcomes

Primary outcomes will be collected at baseline and weekly over a period of 6 months of intervention through an online, self-reported electronic questionnaire, created by the research team. The physical activity intervention group will receive a reminder every week through the web app with a brief questionnaire related to the primary outcomes. The standard care group will receive a SMS reminder every week with a link to the same questionnaire. Secondary outcomes will also be collected electronically at baseline, 6 and 12 months. Both groups will fill out the same questionnaire through the same system. The electronic version of the baseline questionnaires and the weekly questionnaire were created by the research group and will be hosted by the University of Sydney.

#### Primary outcomes

##### Care-seeking associated with LBP

*C*are-seeking will be assessed at baseline and weekly over a 6- month period through a specifically designed electronic questionnaire. The participant will be able to register information related to care-seeking such as any visits to a health practitioner (i.e. general practitioner, physiotherapist, chiropractor, etc), type of pain self-management (i.e. heat pack, bed rest, hot shower, etc), and medication intake (i.e. type of medication taken).

##### Pain levels

Pain levels will be assessed with the numerical rating scale (NRS) [28]. The NRS is an 11-point scale ranging from 0 to 10, where 0 defines absence of pain and 10 describes unbearable pain [28]. The weekly electronic questionnaire developed for the trial will gather average weekly pain levels based on the NRS [28].

##### Disability

Functional disability will be assessed with the Roland–Morris Disability Questionnaire (RDQ) [29]. It consists of 24 questions focusing on normal activities of daily life. Each affirmative answer corresponds to 1 point and the final score is determined by the total number of points. Total score ranges from 0 to 24 and higher scores indicate higher disability. Scores above 14 indicate severe impairment [30]. The weekly electronic questionnaire will also gather weekly disability impairment based on the RDQ.

### Secondary outcomes

Secondary outcomes will include:

*Physical activity:* Self-reported physical activity will be measured using the International Physical Activity Questionnaire (IPAQ) [31]. Objectively measured physical activity will be assessed over a 7-day period using a matchbox-sized accelerometer (*Actigraph* GT3X+). Participants will be instructed to wear the accelerometer on the right hip, attached via an adjustable elastic belt, for seven consecutive days during waking hours (except during water activities or bathing). Activity counts per second will be collected at a sampling frequency of 30 Hz and reintegrated to 60-second epochs for data analysis. The mean counts/minute/day ActiGraph measure will be computed as the total counts accumulated in a valid day divided by the wear time of that day. To be considered as a valid day for analysis, ActiGraph wear time must include 10 h or more. Periods of 60-minutes or more of consecutive zeros (indicating non-use) will be considered as “off-time”. Participants will receive a diary to record all their activities on their waking hours during the 7 days that they will be wearing the *Actigraph.* Accelerometer data will be manually checked against participant diary to verify wear time and erroneous data will be excluded prior to analysis.

*Goal attainment* will be assessed using the Goal Attainment Scale (GAS) [27]. Two long-term goals will be defined by the participant at trial entry GAS is a method used to evaluate interventions according to the attainment of a number of participant-specific goals. In effect, each participant has his/her own outcome measure but this is scored in a standardised way to allow proper statistical analysis. Traditional standardised measures include a standard set of tasks (items) each rated on a standard level. In GAS, tasks are individually identified to suit the participant, and the levels are individually set around their current and expected levels of performance [32]. According to a recent systematic review, GAS delivers reliable and valid scores when employed as an outcome measure in working age and older people within a physical and neurological rehabilitation environment [33].

### Sample size calculation

The sample size for this trial was determined in order to detect a 2-point difference between groups on the pain intensity outcome measured by the Pain Numerical Rating Scale, assuming a standard deviation of 1.9 points [34]. According to a study conducted by J. T. Farrar et al. [35], in a randomized controlled trial a raw change of 1.74 and a percent change of 27.9 % on a 0–10 pain intensity scale is a clinically important improvement [36].

The following specifications were used: statistical power of 80 %, alpha of 5 %. Anticipating maximum loss to follow-up of 35 % [37] the calculated target sample size is 68 patients (34 participants per group). As this is a pilot study, it will investigate the feasibility of conducting a randomized controlled trial (RCT) testing an innovative physical activity management strategy to prevent decline in clinical outcomes following conservative treatment for people with chronic LBP. Findings from this trial will inform on the feasibility, effect size and design of a large multi-centred RCT.

### Statistical analysis

The effect of treatment will be separately analysed for each outcome using linear mixed models with time as a repeated factor, group as a fixed factor and participants as a random factor. The coefficient of the group x time interactions will provide estimates of the effects of interventions over time. Between-group differences in mobility-related goal attainment, at 6 months after randomisation, will be analysed with ordinal regression. To aid interpretation of goal attainment, the scores will also be dichotomised (goal met versus goal not met), and odds ratios calculated. Accelerometer data will be processed using ActiLife 6 software. Acceptable wear time will be set a priori and defined as 4 days or more of 10 h or more per day. All analyses will be performed by intention-to-treat and blinded to treatment group. Potential covariates that will be investigated are baseline pain and disability levels, number of previous treatments, symptom duration, co-morbidities, age and socioeconomic status.

### Process evaluation: qualitative study

Several face to face semi-structured interviews will be conducted with a minimum of 20 participants from the physical activity intervention group, at 1 and 6 months after study enrolment, in order to understand the experiences and attitudes of participants with regard to undertaking the intervention. Participants will be judgmental sampled to obtain a range of demographic data including gender, age and physical activity level. Participants will be interviewed about perceived advantages and disadvantages of the intervention, motivation, self-efficacy and confidence, beliefs about physical activity. The main facilitators and barriers of the intervention will be identified using thematic analysis.

### Ethics

The trial includes key methodological features to minimise bias in controlled trials: randomisation, concealed allocation, specification of eligibility criteria, blinded outcome assessment, blinded analysis, and intention-to-treat analysis. Data will be stored in spreadsheets and transferred to appropriate statistical software for analysis by an investigator blinded to group allocation. Spreadsheets will be regularly scrutinised for omissions and errors. Data will be stored and accessed as per the University of Sydney ethics requirements.

This protocol was registered at the Australian New Zealand Clinical Trials Registry (ACTRN12615000189527) and was prospectively approved by the Human Research Ethics Committee from the South Western Sydney Local Health District (Local HREC reference 15/015).

## Discussion

This RCT will represent a major advance in the field because it will investigate a new model of care to prevent clinical decline in patients who have received the benefits of conservative treatment for chronic LBP. Patients who experience recurrence of LBP after treatment are prone to seek additional care in the form of physiotherapy, medication, and attending emergency departments. This intervention aims to empower patients to self-manage their LBP as well as to prevent back pain recurrence, disability and reduce care seeking by offering patients a contemporary patient-centred physical activity program with the support of mHealth technology. This new model of care is based on a model used in a funded NHMRC trial combining physical activity promotion and fall prevention in older people [38] which is managed by co-investigator Tiedemann. This is an innovative approach of translating knowledge from health fields and a successful model will be translated to a population recovering from LBP that seeks care through public and health private systems. The impact on reducing the current yearly $1 billion treatment costs for LBP could be substantial. The outcomes of this program of research will have immediate clinical practice implications. If effective, this new model of care has the potential to be implemented in the management of other chronic conditions that would benefit from increased physical activity participation, such as osteoarthritis, heart disease and diabetes. The results of this trial will be published once the study is concluded.
